# Interaction of Metals, Menopause and COVID-19—A Review of the Literature

**DOI:** 10.3390/biology12030350

**Published:** 2023-02-22

**Authors:** Tomasz Męcik-Kronenberg, Aleksandra Kuć, Daria Kubik-Machura, Klaudia Kościelecka, Lidia Radko

**Affiliations:** 1 Department of Pathomorphology, Faculty of Medical Sciences in Zabrze, 3 Maja St. 13, 41-800 Zabrze, Poland; 2 Department of Preclinical Sciences and Infectious Diseases, Faculty of Veterinary Medicine and Animal Sciences, Poznan University of Life Sciences, Wolynska St. 35, 60-637 Poznan, Poland

**Keywords:** menopause, metals, COVID-19, connection

## Abstract

**Simple Summary:**

Identified in 2019, the novel coronavirus SARS-CoV-2 causes the disease entity named COVID-19 (coronavirus disease-19). Similarly, in 2003, SARS-CoV that causes severe acute respiratory syndrome (SARS) was identified. This has raised concerns about coronaviruses as disease-causing agents in both humans and animals. This literature review provides an extensive discussion of the relationship between metal exposure and menopause in women with a particular focus on the impact of menopause on the course of COVID-19 and the relationship between metals and SARS-CoV-2 infection in humans.

**Abstract:**

A growing number of reports point to the possible role of environmental factors in determining the age of onset of menopause. Specific metals, such as mercury, cadmium, arsenic and lead can lead to fertility disorders, to endocrine dysregulation, and in addition, their high blood concentrations correlate with the onset of menopause. Changing concentrations of hormones in the blood during this period of a woman’s life can also have an impact on SARS-CoV-2 infection, and excessively high or low levels of metals may also be an important predictor for the course of COVID-19. Postmenopausal women are exposed to greater risk of serum biochemical changes, and with the possibility of nutritional disturbances, particularly involving trace minerals, the risk of age-related diseases is very high during this period. These adverse changes in serum trace minerals should be taken into consideration for the early diagnosis and prevention of menopause-related diseases. Dietary supplementation may be necessary, especially where levels are significantly reduced. We performed a manual search of scientific articles cited in major electronic databases (PubMed, EMBASE, Web of Science and Google Scholar) in November 2022 to identify studies relevant to the relationship between metals, COVID-19 and menopause. The effects of metals on the course of menopause is a broad topic and should certainly still be a subject of research, due to, among other things, continuing environmental pollution and the use of metals in many areas of life.

## 1. Introduction

Menopause is the final stage of ovarian physiology in women and represents the time of transition from reproductive to post-reproductive life. Complete depletion of ovarian follicle resources leads to loss of reproductive function [1]. Many scientific publications are available indicating the association of menopause with the incidence of various diseases in many systems, among which we distinguish mental health disorders [2], cardiovascular disease [3,4,5], osteoporosis [6,7], type 2 diabetes [8,9,10] and other chronic conditions [11]. Specific metals such as mercury, cadmium, arsenic, and lead can lead to fertility disorders, endocrine disruption, and additionally, their high blood concentrations correlate with the onset of menopause [12,13,14,15,16,17,18,19].

Moreover, overall mortality has been associated with an earlier age of onset of menopause [20,21,22]. The beginning of research into the effects of environmental pollutants has provided evidence of their effects at different times in a woman’s life. An increasing number of reports point to the possible role of environmental factors in determining the age of onset of menopause [23,24,25].

Relatively few publications address the disruption of the endocrine system by metals to which the general population is exposed through ambient air, water, food and stimulants such as tobacco [26]. Inconsistent results can be seen when analyzing studies on the relationship between exposure to certain metals and the age of ovarian extinction. Therefore, the correlation of women’s exposure to metals with ovarian aging still remains unexplained [27,28,29,30,31].

Changing hormone concentrations in the blood during menopause may have an impact on SARS-CoV-2 infection, and metals are another factor on which there are scientific reports. Excessively high or low levels of metals may be a significant predictor of the course of COVID-19. This is a viral infectious disease that mainly attacks the respiratory tract. It can manifest similar to the common cold, but in more severe cases, it causes complications such as respiratory failure and pneumonia. Although three years have passed since the first case of the virus, the disease is still an important medical problem.

The following literature review provides a comprehensive discussion of the relationship between metal exposure and menopause and identifies the importance of metals in relation to the extinction of ovarian function. The review focuses on the normal picture of menopause including the course, symptoms, and their alleviation, as well as the effects on various systems. We also analyze the impact of menopause on the course of COVID-19 and the relationship of metals to SARS-CoV-2. This paper summarizes recent scientific reports with the purpose of addressing these topics. 

## 2. Materials and Methods

An extensive manual search of major electronic databases (PubMed, EMBASE, Web of Science and Google Scholar) was conducted in November 2022 to identify relevant studies published on metal compounds, COVID-19 and menopause. No lower date limit was specified. Articles were limited to those published in English and Polish. Multiple search terms were used, including “menopause”, “metals”, “COVID-19”, “menopause and lead”, “menopause and arsenic”, “menopause and cadmium”, ”menopause and mercury”, “menopause and molybdenum”, ”menopause and COVID-19”, and “metals and COVID-19.” The articles were analyzed first by title, then by abstract, and finally by full text. All the selected articles were the most relevant available for this review.

## 3. Results

### 3.1. Menopause—Course, Symptoms and Their Alleviation

Menopause undoubtedly represents a major change and an important aspect of every woman’s health and life. The age of onset of menopause is not strictly defined. It is influenced by many different factors such as lifestyle (understood as diet, stimulants, and physical activity), as well as socioeconomic status, race, genetic factors, variables related to childbirth, the menstrual cycle [32,33] or exposure to metals and their compounds [28,30,31,34], among others. It most often occurs in the age range of 45–55 years. Menopause can be divided into three sub-periods including premenopause, perimenopause and postmenopause, the time frame of which is also difficult to define. Despite the difficulty in estimating the specific time of onset of menopause, the changes in a woman’s body are characteristic and consecutive, and the symptoms are consistent. During premenopause, there is an increase in folliculotropic hormone (FSH) and decreases in estradiol, testosterone and androstendione. Ovarian granulosa cells also secrete less inhibin. In contrast, estradiol and, to a lesser extent, estrone concentrations decrease during postmenopause. Luteotropic hormone (LH) and FSH concentrations are elevated, and prolactin concentrations may or may not be slightly reduced. After menopause, sex steroid binding globulin (SHGB) is reduced and testosterone concentrations are consequently elevated [35]. These changes manifest themselves by causing disruption of the menstrual cycle until it stops altogether. Changing hormone concentrations also cause many other symptoms such as hot flashes, night sweats, volatility and lowered mood, often leading to depression, impaired ability to concentrate and difficulty remembering, insomnia, dizziness and headaches, fatigue, as well as palpitations or stress incontinence. Estrogen deficiency is thought to be the main cause of these symptoms [36].

Insufficient levels of hormones from this group affect virtually every system of a woman’s body, causing, among other things, a decrease in the collagen content of the skin, resulting in its thinning and loss of elasticity [37]. In the skeletal system, in turn, it lowers bone mineral density, leading to osteoporosis [6,7,38]. Estrogen deficiency has also been shown to have a significant effect on the occurrence of cardiovascular conditions based mainly on the deterioration of endothelial function [5,39]. It should be mentioned that ischemic heart disease is currently the leading cause of death occurring in postmenopausal women [40]. Estrogen deficiency is also responsible for the onset of genitourinary changes associated with atrophy that are manifested by dryness, lack of elasticity, pain during intercourse, and an increased need to urinate [41].

Further links between hormonal changes in menopause and predisposition to the disease in question relate to the events of recent years, when the COVID-19 pandemic swept the world. The impact of menopause on the severity of the SARS-CoV-2 virus disease has begun to be considered [42]. Ding et al. (2021) and Liu et al. (2020) showed that menopause was an independent risk factor in terms of prolonged hospitalization and a more severe course of the disease [43,44]. The authors also showed in a study of 78 women from Wuhan that estradiol negatively correlates with disease severity. However, subsequent authors analyzing the topic have not confirmed the effect of menopause on the course of infection, so the correlation is still not fully confirmed [42,43,44].

The troublesome symptoms of menopause undoubtedly affect the comfort of women’s lives. The alleviation of menopausal symptoms is mainly based on the use of hormone replacement therapy that consists of taking phytoestrogens, selective estrogen receptor modulators and bisphosphonates [35].

As estradiol, which belongs to the estrogen group, plays an important role in an adequate and effective immune response, the role of hormone replacement therapy in menopausal women becomes all the more important and recommended in the era of COVID-19 [45].

### 3.2. Metals and Menopause

Women’s risk of experiencing neurological, psychiatric, cardiovascular and osteoporotic diseases increases with earlier onset of menopause [11]. On the other hand, delayed menopause can increase the risk of ovarian and breast cancer due to prolonged exposure to estrogen [46]. Few epidemiologic studies have examined associations between exposure to metals and age at menopause [23,25].

There are many factors that affect the timing and course of menopause. The metals to which we are exposed in every walk of life are one of them. Heavy metals, i.e., arsenic, cadmium, mercury and lead, are significant environmental pollutants, and their toxicity poses a serious threat to human, animal and environmental health. Heavy metals enter the environment naturally and as a result of human activities. The literature describes the association of menopause with exposure not only to heavy metals, but also to metals, i.e., calcium, manganese, molybdenum, and zinc [47,48,49,50,51,52]. In the following sections, we discuss the effects of specific metals on menopause and the data obtained from the literature are summarized in Table 1.

#### 3.2.1. Arsenic

Arsenic (As) is a natural element that can be found in the environment. Food is the main source of arsenic for people. The highest levels of arsenic in foods can be found in seafood, rice, rice cereal (and other rice products), mushrooms, and poultry, although many other foods, including some fruit juices, can also contain arsenic.

The relationship between the toxicity and metabolic processes of arsenic and the effect on the age of natural menopause is not clear. Yunus et al. (2014) showed that women with arsenic skin lesions, a diagnostic sign of chronic arsenic poisoning, were 2.1 years younger at menopause compared with women who had not experienced exposure to the metal [29].

In another study, the authors suggested that female adult rats may be less susceptible than males to arsenic neurotoxicity. At menopause, there is a deficiency of endogenous estrogen (E2), which regulates brain-derived neurotrophic factor (BDNF) and hippocampal signaling of bone morphogenetic protein (BMP) [53]. Consequently, the hormone blocks arsenic-induced neuronal dysfunction in females, which can be inhibited under conditions of E2 deficiency, such as in menopause [53]. Similar findings were presented in two papers indicating endocrine disruption, but also arsenic-induced effects on uterine function and structure, resulting in interaction with estrogen receptors and translating into negative health effects in women [54,55]. Pan et al. (2020) observed, on the other hand, higher concentrations of arsenic in the urine of women with primary ovarian failure, which may indicate its negative effects on ovarian reserves, reproduction or the discussed timing of menopause onset [30]. According to some authors, arsenic is associated with earlier onset of menopause [29,34].

#### 3.2.2. Cadmium

Cadmium (Cd) is a metallic element that occurs naturally in the earth’s crust. It can also be released into the environment as a result of human activities. For non-smokers, the main source of cadmium intake is food. Plants, animals, fish and shellfish take up cadmium from polluted environments. However, for smokers, tobacco smoke is an important source of cadmium exposure [26]. One of the few well-described examples of menopausal-related effects of metals is the very painful disease called Itai-itai, which is a combination of osteoporosis, osteomalacia and renal damage caused by consumption of cadmium-polluted rice [26]. Recent data demonstrate mild effects of cadmium on both kidney and bone with present environmental exposure levels. Women may be at greater risk than men because of increased gastrointestinal uptake of cadmium at low iron stores, which is common in women of childbearing age. Thus, improvement of iron status, which often occurs at menopause, has a positive effect on cadmium exposure in the sense that its absorption decreases. Cadmium accumulates in the kidney with a half-life of 10–30 years [26,56]. The health effects appear around menopause, concurrent with the peak in renal cadmium concentrations [26,56].

Cadmium is another of the metals that disrupts the endocrine system [57,58,59]. It has been shown that this metal, by binding to receptor 30 (GPR30), the nuclear estrogen receptor, and indirectly through the low-density lipoprotein receptor and P450 side-chain cleavage, can cause hormone disruption [60,61,62]. Estrogen activity is disrupted by cadmium, which has additional toxic effects on the ovary [56,63,64,65,66,67]. Expression of estrogen receptor alpha mRNA can be increased by estrogen in combination with cadmium, to a greater extent than alone [62]. In turn, other authors have shown that decreasing iron stores in premenopausal women are associated with higher concentrations of cadmium [68,69,70]. It is also worth noting the correlation between increasing FSH and cadmium among peri-menopausal women with BMI ≥ 27 [71]. Other researchers analyzed cadmium exposure and its effects on menopause in a Chinese population. In women living in highly polluted, moderately polluted and control areas, the menopausal age was 47.1, 47.3 and 47.5, respectively, after adjusting for exposure duration. Based on the data compiled, they concluded that in cadmium-polluted areas compared with control areas, the incidence of delayed menopause was slightly increased, also indicating a mimetic effect of the metal on estradiol [72]. Still other authors argue that cadmium’s effects on menopause in women require further study [73,74,75].

#### 3.2.3. Mercury

Mercury (Hg) is one of the most dangerous toxic elements. Mercury is released into the environment from natural and anthropogenic sources. Food is the primary source of Hg exposure for humans. High mercury exposure comes from fish and other sea animals. Increased consumption of fish as a source of dietary proteins and omega-3 fatty acids could raises the risk of methylHg (MeHg) induced health problems [16,17].

Some authors conclude that the association of mercury with early menopause is still unclear [17]. However, other researchers report that mercury concentrations are lower in menopause than in premenopause [16].

#### 3.2.4. Lead

Lead (Pb) toxicity is a subject of interest for scientists due to its effects on plants, animals, and humans. An increase in several Pb-related industrial activities including mining and the use of Pb-containing products, such as agrochemicals, oil and paint etc., can lead to Pb contamination in the environment, resulting in Pb entering the food chain. Being one of the most toxic heavy metals, Pb ingestion via the food chain has proven to be a potential health hazard for humans [13]. A large portion (20–70%) of ingested Pb is absorbed by the human body [13], and about 90% of body lead is localised in bone. There is a significant release of bone lead after menopause in association with the acceleration of bone resorption. Thus, postmenopausal women may be at increased risk of the adverse effects of lead.

Serum ferritin levels are the most optimal laboratory indicator for assessing iron stores as serum ferritin levels correlate with total body iron stores. Sim et al. (2014) reported that blood lead concentrations are higher in menopausal women than in premenopausal women, despite the former having higher ferritin concentrations, which may be due to the metal’s association with estrogen [76]. It has been shown in the literature that the higher concentrations of lead may be due to its mobilization from the skeleton during periods of increased bone demineralization, such as in menopause [28,47]. It is worth mentioning that an earlier onset of menopause has been noted in women occupationally exposed to lead [31]. It has also been noted in women with higher blood lead levels [28,34].

In contrast, Eum et al. (2014) made observations of tibia lead content as an indicator of lead exposure, but found no relationship between age of menopause onset and lead levels in the blood or patella [27].

#### 3.2.5. Calcium

Calcium (Ca) is most commonly associated with the formation and metabolism of bone [51,77,78,79,80]. In the circulatory system, extracellular fluid, muscle, and other tissues, Ca is important for mediating vasocontraction and vasodilatation, muscle function, nerve transmission, intracellular signaling, and hormonal secretion [51,78,78,80]. Deficiency of Ca can result in reduced bone strength and osteoporosis, characterized by fragile bones and increased risk of falling.

Only one study concerning calcium and menopause was found. In Japanese women, higher calcium intake was associated with a higher probability of early onset of menopause [77]. Calcium deficiency and osteoporosis are common in postmenopausal women.

#### 3.2.6. Magnesium

Magnesium (Mg) is a bivalent intracellular cation and has been recognized as a cofactor for more than 300 metabolic reactions in the body [12]. It is important in maintaining normal nerve and muscle function, blood pressure, bone integrity, cardiac excitability, glucose and insulin metabolism [12,79,81]. Magnesium deficiency has been associated with a number of chronic diseases including migraine headaches, hypertension, cardiovascular diseases, and osteoporosis, which are common amongst postmenopausal women [12,79,81].

Due to higher ferritin levels in menopausal women relative to younger women, blood magnesium concentrations are lower in the former [82]. However, studies determining the effect of magnesium on the timing of menopause are lacking.

#### 3.2.7. Molybdenum

Several scientific papers have proven an association between supplementation with mineral-vitamin preparations and consumption of molybdenum-rich legumes and later onset of menopause [34,77,83].

**Table 1:** Comparison of elemental concentrations in blood during premenopausal and menopausal periods. Based on [16,47,48,49,50,51,52,84].

Element Concentration in Blood	Premenopausal Period	Menopause
cadmium	lower	higher
lead	lower	higher
manganese	higher	lower
arsenic	no data	no data
calcium	lower	higher
molybdenum	no data	no data
zinc	higher	lower
mercury	higher	lower

#### 3.2.8. Supplementation

Many of the unpleasant symptoms of menopause can be alleviated with specific hormonal treatments, but women should also take care of a proper diet that provides adequate levels of vitamins and minerals. As far as possible, all the components necessary for the proper functioning of the body should be provided in the most natural form possible. However, this is often not possible, if only because of the climate zone in which women live or the lifestyle they lead. Proper nutrition or supplementation can help protect against the possible consequences of menopause, such as osteoporosis. Table 2 shows the available information on supplementation of metal preparations in menopausal women who have no significant diseases or conditions that may cause deficiencies, such as gastrointestinal bleeding or impaired absorption syndromes, and the effects of supplementation on the bodies of these women.

### 3.3. Metals vs. COVID-19

A study by Skalny et al. (2020) discusses the impact of exposure to toxic metals in the context of the incidence and course of respiratory diseases, including viral diseases, particularly COVID-19 [89]. Farsalinos et al. (2020 a, b) indicate that smoking is a significant factor affecting the incidence of COVID-19. Smokers are at higher risk than non-smokers, but lower risk than past smokers [90,91]. Another factor that may predispose people to an increased risk of more severe COVID-19 and also mortality, is long-term environmental pollution. Airborne pollution in which NO_2_ and O_3_, PM10 (particulate matter with a diameter of 10 μm or less), and PM2.5 (particulate matter with a diameter of 2.5 μm or less) levels were elevated was positively correlated with the occurrence of new cases of the disease, while exceeding PM2.5 standards resulted in an 8% increase in mortality [92,93]. Both cigarette smoke and air pollution are sources of heavy metals. It should be noted that the epidemiological threat related to SARS-CoV-2 has radically changed the mobility of citizens in the world. This dramatic event resulted in significant improvements in air quality in urban regions [94,95]. In the work of Quarato et al. (2017) it has been shown that residents of industrial areas are exposed to higher levels of toxic metals, thereby increasing the risk of many diseases, including cancer [96]. Nuñez et al. (2016) showed a relationship between the increased content of arsenic or chromium in the soil and cancer mortality in particular sexes. Increased arsenic content in the topsoil increased the incidence of colorectal, oral and kidney cancer in the male population. Increased chromium content in the topsoil increased the incidence of breast cancer, upper gastrointestinal cancer and non-Hodgkin’s lymphomas (NHL) in the female population [97]. Based on the above literature data, it can be concluded that environmental pollution has an impact on human health and thus on the course of the COVID-19 disease.

Higher levels of arsenic, cadmium, mercury and lead found in patients are associated with impaired respiratory function and the incidence of respiratory diseases [98]. Reduced forced vital capacity (FVC) and forced expiratory volume in one second (FEV1), dysfunction of mucociliary clearance and dysfunction of the natural barriers of the respiratory system have been observed in people exposed to the listed toxic metals [98]. Analysis of the levels of Hg, Pb and Cd in patients did not show any significant changes in the concentrations of Hg and Pb in patients’ blood. High concentrations of Cd have been found in COVID-19 deaths [98], and the urinary concentrations of tested heavy metals were significantly increased in patients with severe COVID-19 [99]. Significantly higher levels of Cd, Hg and Pb in the urine of patients with severe COVID-19 could be due to kidney damage of toxic-viral etiology. Therefore, heavy metal antagonists, i.e., selenium, zinc, copper, and magnesium, are recommended to at least slightly prevent the adverse effects of this exposure [99]. Current clinical trials will provide interesting data on the relationship of Zn, Cu, Se, vitamins A, D and E in COVID-19 with adverse effects during viral infection. There are still many hypotheses that heavy metal toxicity affects the morbidity, course, complications and mechanisms concerning COVID-19, but they need to be confirmed by further studies [98]. It is nevertheless worth remembering to properly protect oneself from harmful factors by, for example, eliminating smoking [90,91].

However, it remains an open question whether only external factors affect who gets sick and how COVID-19 infection progresses. Jothimani et al. (2020) noted that zinc deficiency increased the length of hospital stay, caused more complications, and worse, increased mortality [100]. Most of the literatures suggests that Zn deficiency or prolonged hypozincemia may be a risk factor for severe COVID-19. These data speak in favor of zinc supplementation in COVID-19 patients. Similar to Zn, hypocalcemia and deficit Se were observed in patients with COVID-19. However, there are no clear data in the literature on the effects of Ca supplementation in patients, but one study provided positive evidence for the importance of Se and Zn supplementation in severe COVID-19 patients (~60.5 of age). Magnesium too has been reported to have a beneficial effect in this disease. Administration of magnesium sulfate is described as having beneficial effects in patients with severe disease [101]. The combined administration of Mg with vitamin D and B12 is associated with a significant reduction in the proportion of patients with clinical deterioration. The level of Mg was investigated in COVID-19 patients, and significantly lowered concentrations of Mg were noted in COVID-19 patients’ blood [97]. Due to the still limited number of studies, the efficacy of magnesium or zinc supplementation cannot be 100% confirmed, but its benefits far outweigh any adverse effects in the face of COVID-19 [102,103,104] and should be considered among vulnerable individuals, including menopausal women.

## 4. Conclusions

Postmenopausal women are exposed to greater risk of serum biochemical changes and possibile nutritional disturbances, particularly in relation to trace minerals. The risk of age-related diseases is very high during this period, and these adverse changes in serum trace minerals should be taken into consideration for early diagnosis and prevention of menopause-related diseases. Dietary supplementation may be necessary, especially where levels are significantly reduced.

The topic of the influence of metals on the course of menopause is broad and should certainly still be the subject of research, due to, among other things, long-term environmental pollution and the use of metals in many areas of life. Reports on the effects of metals on the timing and course of menopause, and the effects of metals on the course of COVID-19 are sparse, hence the need for further studies on a larger scale to draw firm conclusions.

There is a need for supplementation, not only with calcium but also with zinc or selenium, in menopausal women to alleviate the clinical symptoms of COVID-19 and the toxic effects of metals. An important topic is the implementation of hormone replacement therapy in postmenopausal women in the era of the COVID-19 pandemic, which should be more widely studied as having a positive impact on the course and severity of the infection. We hope that the information in the article will be useful to those interested in the effects of heavy metals on menopause and their impact on COVID-19, as well as therapeutic strategies against SARS-CoV-2 in older menopausal women.

## Figures and Tables

**Table 2:** Metal supplementation in menopause. Based on [78,79,80,81,84,85,86,87,88,89,90,91].

Metal	Assessment of the Need for Supplementation and Its Effects.
Iron	No supplementation needed.
Zinc	Possible interaction with calcium in maintaining adequate bone density. Better results than in calcium supplementation alone. Further research needed.
Calcium	Need for supplementation to prevent osteoporosis.
Manganese	Possible interaction with calcium in maintaining adequate bone density. Better results than in supplementation with calcium alone. Further research needed.
Copper	Possible interaction with calcium in maintaining adequate bone density. Better results than in supplementation with calcium alone. Further research needed.
Magnesium	Further research needed.
Chromium	No information available.
Potassium	Possible action in reducing bone resorption and increasing the rate of bone formation. Further research needed.

## Data Availability

Not applicable.
